# The complete mitochondrial genome sequence of *Squalidus multimaculatus* (Cypriniformes: Cyprinidae)

**DOI:** 10.1080/23802359.2017.1347900

**Published:** 2017-07-11

**Authors:** Seul Yi, Sang-Hwa Lee, Taeseo Park, Min-Ho Jang

**Affiliations:** a Animal Resources Division, National Institute of Biological Resources, Incheon, Republic of Korea;; b Department of Biology Education, Kongju National University, Kongju, Korea;; c Graduate Program in Cellular Biology and Genetics, College of Medicine, Chungbuk National University, Cheongju, Republic of Korea

**Keywords:** Mitogenome genome, Cyprinidae, *Squalidus multimaculatus*

## Abstract

This study determined the complete mitogenome sequence of *Squalidus multimaculatus* (Cypriniformes: Cyprinidae). The mitogenome of *S. multimaculatus* is 16,597 bp in length, including 13 protein-coding genes, 22 transfer RNAs, 2 ribosomal RNA genes, and a non-coding control region. NCR is 925 bp in length, is located between tRNA^pro^ and tRNA^phe^. The overall base composition of *S. multimaculatus* is 27.9% for A, 18.9% for G, 25% for T, 28.2% for C, with a slight AT bias (52.9%). These results will provide the data required for phylogenetic studies of the *Squalidus* species.

The genus *Squalidus* (Cypriniformes: Cyprinidae) fishes are constituted of 18 valid species (Froese and Pauly 2017), found widely distributed in East Asia. Of these, 4 species are distributed in South Korea (Banarescu and Nalbant 1973). The genus *Squalidus* can be classified by morphological characteristics such as length of the lip beard, size of eye, height of the body depth, and presence of side spots (Kim and Lee 1984). *Squalidus multimaculatus* is clearly distinguished by black spots on the side of the body. This species is endemic in Korea, and is solely distributed in rivers flowing to the East coast (Kim and Park 2007). Due to the insufficient ecological and genetic information available (Kim 1997), this study undertook to determine the mitochondrial genome sequences of *S. multimaculatus*.

A specimen of *S. multimaculatus* was collected from the Baebong Stream, Myungpa-ri, Hyunae-myun, Goseong-gun, Gangwon-do, South Korea (38.5445N, 128.4030E). Voucher specimen was housed at National Institute of Biological Resources (NIBRP0000037107). Following 3 steps of mtDNA enrichment (mitochondrial isolation, mtDNA extraction and amplification of mtDNA), the mtDNA was extracted and sequenced on the Illumine HiSeq2000 platform, using gene annotation methods described by Song et al. (2016).

The complete mitogenome of *S. multimaculatus* (GeneBank accession no. KX49560) was 16,597 bp in length, including 13 protein-coding genes (PCGs), 22 transfer RNAs (tRNA), 2 ribosomal RNA (rRNA) genes, and 1 non-coding control region (NCR). The NCR was 925 bp in length, located between the tRNA^pro^ and tRNA^phe^. The start codon and termination codon of the 13 protein-coding genes are as follows. The start codon of the 12 protein-coding genes is ATG, and the start codon of COXI is GTG. Six genes (COXI, ATP8, ATP6, NAD4L, NAD5, NAD6) end in the TAA termination codon. Three genes (NAD4, NAD1, NAD2) contain TAG as the stop codon, whereas four genes (COXII, COXIII, NAD3, Cyt b) share the incomplete stop codon T. The tRNA sequence length ranges from 68 to 76 bp, and has the shape of a three-leafed clover structure. Two rRNAs are 12S rRNA (959 bp) and 16S rRNA (1,685 bp). The overall base composition of *S. multimaculatus* was evaluated as 27.9% A, 18.9% G, 25% T, 28.2% C, and with a slight AT bias of 52.9%.

To examine the phylogenetic position of *S. multimaculatus*, the mitochondrial genome sequences of 16 species in 7 genera belonging to Cyprinidae were mined from GeneBank. The phylogenic tree was constructed using the maximum-likelihood analysis. A close relationship was seen with the two species of *S. japonicus coreanus* and *S. gracilis gracilis* (Figure 1). Though populations of *S. multimaculatus* are abundant, the habitat is limited, making it necessary to establish more information for this species. We envisage that our study results will contribute to elucidate the phylogenetic relationship among all the species of the *Squalidus* group.

**Figure 1.**
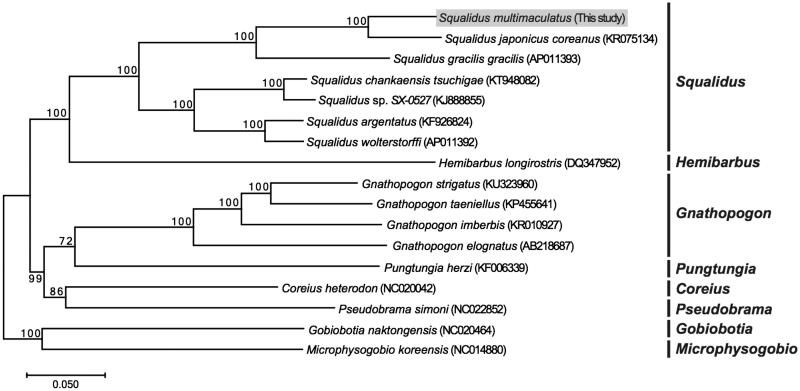
A phylogenetic tree of *Squalidus multimaculatus* with other cyprinid species base on complete mitogenome sequences using maximum-likelihood analysis. Bootstrap replicates were conducted 1000 times.
